# A double-edged sword: unpacking the functional heterogeneity of mobile social media use and its differential associations with exercise identity and social-media-specific sedentary time among older adults

**DOI:** 10.3389/fpubh.2026.1902864

**Published:** 2026-09-23

**Authors:** Ningning Liu, Guofeng Zhang, Xiaoyuan Han, Ning Gai, Yuan Gao, Wenjun Wang, Xue Yao

**Affiliations:** 1 School of Basic Medical Sciences, Jining Medical University, Jining, China; 2 Jining Polytechnic, Jining, China; 3 Faculty of Psychology, Southwest University, Chongqing, China; 4 Key Laboratory of Cognition and Personality, Ministry of Education, Chongqing, China; 5 Department of Bone and Joint, The First People's Hospital of Jining City, Jining, China; 6 Weifang Nursing Vocational College, Qingzhou, Shandong, China; 7 School of Public Health, Shandong Medical and Pharmaceutical University, Yantai, China

**Keywords:** dual-path model, exercise identity, mobile social media functions, older adults, sedentary time

## Abstract

**Background:**

As digital life prevails among older adults, this study investigates how mobile social media functions affect their exercise identity and social-media-specific sedentary time.

**Methods:**

Using the S-O-R framework, a dual-path model was tested with SEM (AMOS 20.0) on data from 452 older adults in China.

**Results:**

Overall, mobile social media functions were not significantly associated with exercise identity or social-media-specific sedentary time. However, specific functions showed divergent effects: information retrieval (*Z* = 6.06, 95% CI [0.14, 0.27]), online guidance (*Z* = 3.32, 95% CI [0.08, 0.57]), and social comparison (*Z* = 2.44, 95% CI [0.05, 0.30]) positively predicted exercise identity, whereas video media and personalized algorithms negatively predicted exercise identity (*Z* = -1.32, 95% CI [−0.57, −0.04]) and positively predicted social-media-specific sedentary time (*Z* = 2.42, 95% CI [0.17, 1.01]). Perceived value mediated the effects of attention and interaction (*Z* = 2.56, 95% CI [0.009, 0.05]) and recording (*Z* = 3.30, 95% CI [0.03, 0.12]) on exercise identity. Immersive experience mediated the effect of video media and personalized algorithms on social-media-specific sedentary time (*Z* = 1.41, 95% CI [0.006, 0.19]).

**Conclusion:**

Mobile social media exerts dual effects on older adults’ exercise identity. Video media and personalized algorithms are key risk factors for social-media-specific sedentary time, while perceived value and immersive experience mediate distinct pathways.

## 1 Introduction

Global population aging, together with the rapid spread of digital technology, is reshaping the lives of older adults (1). Mobile social media is no longer exclusive to the young. It has become an important digital tool for older adults to access health information, maintain social connections, and engage in leisure activities (2). Adults aged 55 and above are now the fastest-growing group of social media users worldwide. Older adults have become a key driver of social media user growth (3).

However, the link between mobile social media and health-related outcomes in older adults is not uniform. Some functions, such as information retrieval and social comparison, may provide cognitive and social resources for constructing exercise identity (4, 5). Algorithm-driven immersive functions, in contrast, may influence exercise activity by extending use time (6, 7). This two-sided pattern suggests that treating social media use as a single aggregated variable may obscure essential differences in how distinct functions affect health.

Previous studies have often examined the association between social media and health behaviors in older adults at the level of overall use time or platform type (1). When different functions are combined into a single measure, their effects may cancel each other out statistically. This may explain the mixed findings in the literature—some studies report that social media use is linked to higher exercise activity levels (3), while others find it is closely associated with increased sedentary time (1). This inconsistency may largely stem from methodological limitations related to construct aggregation. In recent years, some scholars have begun to examine the independent roles of specific social media functions (5). Yet few studies have systematically compared how multiple functions relate to different health outcomes within a single analytical framework. The underlying psychological mechanisms also remain under explored.

This study draws on the Stimulus-Organism-Response (S-O-R) theory as its conceptual framework (8). The theory proposes that environmental stimuli influence an individual’s internal state, which in turn shapes behavioral responses. In the context of mobile social media, different platform functions represent distinct environmental stimuli. Functions such as information retrieval and online guidance primarily activate users’ overall evaluation of the platform experience, namely perceived value (4, 5). This may promote health-related self-concept and facilitate the internalization of exercise identity. Video media and personalized algorithms, by contrast, are more likely to induce immersive experience (6, 7). This psychological state may weaken self-regulation and prolong unintentional platform-specific sedentary time. Thus, perceived value and immersive experience represent two different psychological mechanisms that may link functions to divergent pathways.

Based on this theoretical logic, this study proposes a dual-pathway association model. It examines how mobile social media functions relate to both exercise identity and social-media-specific sedentary time in older adults. It also tests the mediating roles of perceived value and immersive experience in these relationships (4). By deconstructing functions and clarifying mechanisms, this study aims to move beyond a simple “good versus bad” narrative about social media. It seeks to provide precise evidence for optimizing age-friendly digital health platforms.

## 2 Materials and methods

### 2.1 Participants

This study adopted a cross-sectional survey design. Shandong province was selected as the survey site. Shandong has one of the largest aged populations in China. Its aging profile and the pattern of digital media use among older adults are representative of the national context.

A two-stage mixed non-probability sampling strategy was used. The first stage employed purposive sampling. This was designed to ensure regional heterogeneity through geographic stratification. The second stage used snowball sampling. This was intended to compensate for the under-coverage of non-community-active older adults in the first stage.

In the first stage, two community senior activity centers were selected from each of the four cardinal directions (east, west, south, north) of Shandong Province. These served as initial recruitment sites. Local community or village committees provided referrals. Potential participants were contacted by phone or through in-home visits arranged with the assistance of community health service centers.

After the first stage was completed, the second stage began. Participants who had already been enrolled and met the age criteria were asked to recommend other eligible older adults in their social networks who were not active in community settings. This snowball approach expanded the sample coverage.

The entire survey was conducted from March to May 2024. Trained graduate students served as survey administrators. Before distributing the questionnaires, they explained the purpose of the study to each participant. Written informed consent was obtained from all participants.

Inclusion and exclusion criteria were as follows. Inclusion criteria: (1) aged 60 years or older; (2) active mobile social media users, defined as using at least one mobile social media platform daily for 30 min or more over the past 3 months; (3) cognitively capable of providing informed consent and able to complete the questionnaire independently or with minimal assistance. Exclusion criteria: (1) cognitive impairment preventing informed consent; (2) severe visual or hearing impairment preventing independent smartphone use; (3) non-users of mobile social media; (4) current residents of nursing homes or hospitalized patients.

A total of 600 questionnaires were distributed. After careful screening, 148 were excluded. The reasons for exclusion were: completion time under 3 min (*n* = 11), patterned or careless responses (*n* = 34), age ineligibility or non-users of mobile social media (*n* = 103). The final sample consisted of 452 older adult mobile social media users. The effective response rate was 75.33%.

The mean age of the sample was 67.47 ± 6.04 years. There were 202 males and 250 females. In terms of education, 275 participants had a high school education or below, 156 had a bachelor’s degree, and 21 had a postgraduate degree. Regarding living arrangements, 127 lived alone and 325 lived with others. The frequency of mobile social media platform use is presented in Table 1.

**Table 1:** Frequency of mobile social media platforms used by participants.

Mobile social media platform	Frequency	Percentage	Case percentage
WeChat	248	24.30%	54.90%
Rednote	92	9.00%	20.40%
Weibo	75	7.30%	16.60%
TikTok (Douyin)	248	24.30%	54.90%
Bilibili	80	7.80%	17.70%
QQ	95	9.30%	21.00%
Kwai (Kuaishou)	103	10.10%	22.80%
Other	80	7.80%	17.70%

This was a multiple-response question. Percentage = proportion of total responses (*N* = 1,021). Case percentage = proportion of participants (*N* = 452).

We conducted an *a priori* power analysis using the R package semPower (9). The model had 1,190 degrees of freedom. The minimum sample size required was 30. Our effective sample was 452 participants. This exceeded the minimum requirement. Thus, statistical power was sufficient. The study was approved by the Ethics Committee of Jining Medical University (JNMC-YX-2024-075). All participants provided written informed consent before enrollment. The study was conducted in accordance with the Declaration of Helsinki.

### 2.2 Theoretical model of S-O-R

Mobile social media have rich and diverse functions, such as information retrieval, goal setting, social comparison, video media, personalisation algorithms, attention interaction, and product services (10, 11), which expand the boundaries of older users’ health experience. Still, they may have a double-edged sword effect on older adults’ health behaviors (12, 13). Based on the S-O-R theory, this study constructs a dual-path model of the impact of mobile social media features on older users’ perceptions and experiences (Figure 1), explores how they promote health behavior change and provides guidance for healthy platform development and user behavior management. In the era of mobile social media, perceived value becomes the core of users’ motivation and needs, influencing their behavioral decisions, and in promoting exercise identity, perceived value serves as an intermediary, highlighting its criticality. Although the immersive experience satisfies social and informational needs, it may blur the sense of time, weaken self-regulation, and prolong sedentary time due to the state of “flow,” which may be detrimental to health. In this model, H1 indicates the predictive effect of mobile social media features on exercise identity, H2 indicates the predictive impact of mobile social media features on social-media-specific sedentary time, H3 indicates the mediating role of perceived value between mobile social media features and exercise identity, and H4 indicates the mediating role of immersive experience between mobile social media features and social-media-specific sedentary time.

**Figure 1:**
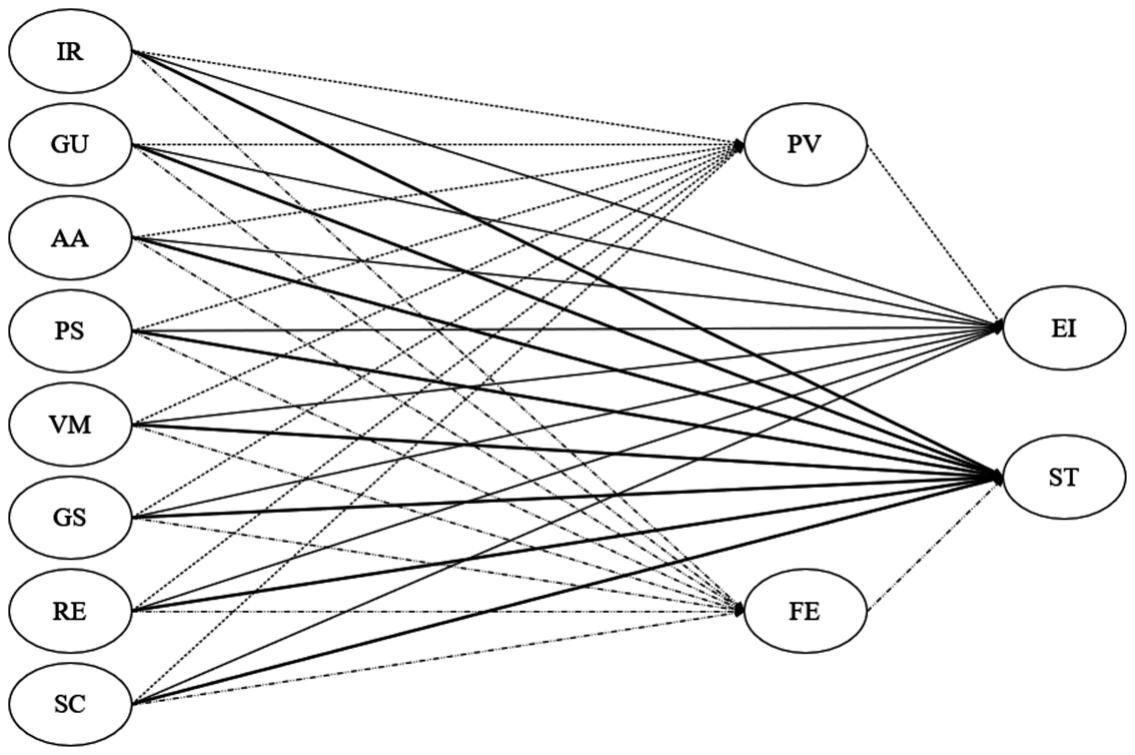
Dual-path hypothetical model diagram. IR for information retrieval; GU for online guidance; AA for attention and interaction; PS for product service; VM for video media and personalization algorithms; GS for goal setting; RE for recording; SC for social comparison; PV for perceived value; FE for flow experience; EI for exercise identity; ST for sedentary time. The solid line at 1pound represents the H1; the solid line at 2 pound represents the H2; a dotted line with squares represents H3; and a dashed line containing a dash, a dot and another dash represents H4.

## 3 Research tool

### 3.1 Mobile social media features

The Mobile social media features scale draws on studies by Anderson and Cychosz, Lee et al., and Sin and Kim (12–14). The scale was measured on a 5-point likert scale and Includes 8 constructs of information retrieval, online guidance, attention and interaction, product service, video media and personalisation algorithms, goal setting, record keeping, social comparison, and a total of 26 questions on “how mobile social media allows me to access useful workout information and knowledge”, and “how mobile social media can provide me with online instruction from my trainer”. The McDonald’s Omega (ω) for each construct of the scale ranged from 0.90 to 0.93, indicating that the scale has good reliability.

### 3.2 Perceived value

The concept of perceived value scale was derived from Sweeney’s Perceived Value Scale (15), which employs a 5-point likert scale encompassing three dimensions: informational value, emotional value, and social value. The scale includes statements such as “The content of the information accessed in mobile social media is accurate” and “Using mobile social media to obtain information makes me feel happy,” along with a total of 12 items. The McDonald’s Omega (ω) coefficient for the scale was calculated to be 0.96, suggesting that the scale demonstrates strong reliability.

### 3.3 Flow experience

The flow experience scale was derived from the works of Chang et al. (16, 17). The measurement employed a 5-point likert scale, which included statements such as “When using mobile social media, I feel that time passes by exceptionally fast” and “When using mobile social media, all my attention is focused on this matter,” among others, culminating in a total of four items. The McDonald’s Omega (ω) coefficient for the scale was calculated to be 0.89, suggesting that the scale demonstrates strong reliability.

### 3.4 Exercise identity

The assessment of exercise identity scale was derived from research conducted by Anderson and Cychoszet et al. (18), utilizing a 5-point likert scale comprising four items. These items include statements such as, “After utilizing a mobile fitness application, I would dedicate a significant amount of time to engaging in exercise activity in my life,” and “After using a mobile fitness application, those in my social circle perceive me as a regular participant in physical exercise.” The McDonald’s Omega (ω) coefficient for this scale is 0.91, suggesting that the scale demonstrates strong reliability.

### 3.5 Social-media-specific sedentary time

The social-media-specific sedentary time scale is defined according to supported criteria (19), and the scale was constructed by integrating findings from the research conducted by Washburn et al. (20). A seven-item scale was created to assess sedentary behaviors specifically within the context of mobile social media usage. This scale includes items such as “the amount of time spent on mobile social media daily while sitting, semi-reclining, or reclining” and “the frequency of mobile social media engagement per day while in a sitting, semi-reclining, or reclining position,” among others. The options are measured at intervals of 0.5 h, covering the time range from 0 to 6 h, using a 12-point equidistant scale. The McDonald’s Omega (ω) coefficient for the scale was calculated to be 0.92, suggesting that the scale demonstrates strong reliability.

### 3.6 Statistical analysis

Descriptive statistics, correlation analysis and structural equation modeling were performed using SPSS 27.0 and AMOS 20.0. The relationship between the eight constructs of mobile social media functionality, perceived value, immersive experience, exercise identity, and social-media-specific sedentary time was examined using Pearson correlation analysis, and *p*-values were corrected for multiple comparisons using FDR. There were no missing data in this study. In the structural equation modeling analysis, model fit indices were assessed using χ^2^/df, GFI, AGFI, CFI, NFI, and RMSEA, with the following thresholds: (1) The value of χ2/df should be between 1 and 3; (2) GFI and AGFI are generally greater than 0.90; (3) CFI and NFI are generally greater than 0.90; (4) RMSEA should be generally less than 0.08 (21, 22). The significance of the mediating effect was tested using the bias-corrected Bootstrap method. The number of Bootstrap iterations was set at 5000. If the 95% confidence interval of the Bootstrap does not include 0, it indicates that the parameter estimates are significant; otherwise, it indicates that the parameter estimates are not significant (23). The *p*-value was corrected using FDR for multiple comparisons. The effect size f^2^ is mainly used to measure the degree of change in R^2^ when a predictor variable is removed from the model. The larger the value, the more significant the explanatory contribution of the predictor variable to the endogenous variable (24, 25). According to Cohen’s recommendation (26), the f^2^ values of approximately 0.02, 0.15, and 0.35 represent small, medium, and large effect sizes, respectively.

## 4 Results

### 4.1 Common method bias test

Most of the data collected were self-reported, which may lead to common method bias in the model due to the social desirability effect. Therefore, the study conducted a common method bias test on the model to determine the existence of CMB with a nested competition model. Among them, a one-way CFA analysis yielded χ^2^ = 12788.47, *df* = 1,175 while multifactor CFA yielded χ^2^ = 3362.86, *df* = 1,109. The difference between the two models was Δ *df* = 66, Δ χ^2^ = 9425.61, *p* < 0.001, indicating that the study is not affected by common variance bias. The significance level for testing all research hypotheses was set at α < 0.05.

### 4.2 Model verification

Measurement model testing is required prior to structural model evaluation. In this study, the CFA measurement model variables were corrected and reduced according to Kline’s two-step model (19). Based on the criteria of Hair et al. (20), standardized factor loadings greater than 0.50, component reliabilities greater than 0.60, and mean–variance extracted values greater than 0.50, It shows that all 12 variables in the model have good reliability and convergent validity. (Table 2).

**Table 2:** Summary of validation factor analysis.

Variable	Item	Significance test parameters				Std.	Reliability	Component reliability	Average variance extracted
		Unstd.	S. E.	*Z*	*p*		SMC	CR	AVE
IR	IR1	1.00				0.90	0.81	0.94	0.85
	IR2	1.04	0.03	33.51	***	0.95	0.90		
	IR3	0.97	0.03	30.26	***	0.91	0.83		
GU	GU1	1.00				0.94	0.88	0.94	0.84
	GU2	1.00	0.03	33.72	***	0.92	0.85		
	GU3	0.95	0.03	30.22	***	0.88	0.77		
AA	AA1	1.00				0.89	0.79	0.90	0.79
	AA2	1.07	0.04	27.49	***	0.94	0.88		
	AA3	0.98	0.04	23.56	***	0.84	0.71		
PS	PS1	1.00				0.82	0.67	0.90	0.76
	PS2	1.06	0.05	21.43	***	0.88	0.77		
	PS3	1.10	0.05	21.90	***	0.91	0.83		
VM	VM1	1.00				0.88	0.77	0.91	0.76
	VM2	1.05	0.04	24.06	***	0.88	0.77		
	VM3	0.99	0.04	23.30	***	0.86	0.74		
GS	GS1	1.00				0.80	0.64	0.90	0.74
	GS2	1.21	0.06	20.61	***	0.89	0.79		
	GS3	1.17	0.06	20.63	***	0.89	0.79		
RE	RE1	1.00				0.93	0.87	0.95	0.87
	RE2	0.99	0.03	35.82	***	0.93	0.87		
	RE3	1.00	0.03	36.32	***	0.94	0.88		
SC	SC1	1.00				0.88	0.77	0.91	0.77
	SC2	1.03	0.04	25.56	***	0.91	0.83		
	SC3	0.95	0.04	22.99	***	0.84	0.71		
PV	PV1	1.00				0.73	0.53	0.96	0.66
	PV2	1.06	0.06	17.45	***	0.81	0.66		
	PV3	1.11	0.06	18.42	***	0.85	0.72		
	PV4	1.04	0.06	17.02	***	0.79	0.62		
	PV5	1.09	0.06	17.35	***	0.80	0.64		
	PV6	1.09	0.06	17.82	***	0.82	0.67		
	PV7	1.14	0.06	18.78	***	0.86	0.74		
	PV8	1.11	0.06	17.62	***	0.81	0.66		
	PV9	1.09	0.07	16.47	***	0.76	0.58		
	PV10	1.13	0.06	18.40	***	0.85	0.72		
	PV11	1.15	0.06	18.84	***	0.87	0.76		
	PV12	1.09	0.06	17.60	***	0.81	0.66		
FE	FE1	1.00				0.86	0.74	0.86	0.62
	FE2	0.95	0.04	21.48	***	0.83	0.69		
	FE3	1.01	0.05	22.71	***	0.87	0.76		
	FE4	0.52	0.04	12.03	***	0.54	0.29		
EI	EI1	1.00				0.94	0.88	0.81	0.60
	EI2	0.88	0.05	17.88	***	0.71	0.50		
	EI3	0.85	0.05	15.65	***	0.64	0.41		
ST	ST1	1.00				0.76	0.58	0.91	0.59
	ST2	1.09	0.07	16.07	***	0.74	0.55		
	ST3	1.23	0.07	19.06	***	0.86	0.74		
	ST4	1.11	0.06	18.42	***	0.83	0.69		
	ST5	1.07	0.06	18.84	***	0.85	0.72		
	ST6	0.59	0.05	11.62	***	0.55	0.30		
	ST7	0.96	0.06	16.47	***	0.75	0.56		

***p < 0.001.

The absolute value of skewness in this study ranged from 0.01 to 0.51, which is less than 2.00, and the absolute value of kurtosis ranged from 0.54 to 1.14, which is less than 8.00, indicating that the data in this study conformed to a normal distribution. The AVE method was applied to analyze the discriminant validity, and the square root of the AVE for each variable had to be greater than the correlation coefficient of each pair of variables, which indicated the discriminant validity between the variables. The square root of the AVE for each variable in this study is greater than the standardized correlation coefficients of the other variables, indicating the discriminant validity between the variables. (Table 3).

**Table 3:** Means, standard deviations and correlation analysis of variables.

Variable	M	SD	|S|	|K|	IR	GU	AA	PS	VM	GS	RE	SC	PV	FE	EI	ST
1. IR	2.97	1.21	0.06	1.14	***0.92***											
2. GU	2.90	1.15	0.01	1.10	0.75	***0.92***										
3. AA	3.14	1.10	0.27	0.94	0.61	0.48	***0.89***									
4. PS	3.28	0.97	0.29	0.54	0.65	0.75	0.38	***0.87***								
5. VM	3.12	1.04	0.03	0.89	−0.70	−0.82	−0.47	−0.69	***0.87***							
6. GS	2.90	1.10	0.14	1.10	0.63	0.49	0.72	0.48	−0.48	***0.86***						
7. RE	2.96	1.19	0.06	1.12	0.76	0.69	0.71	0.56	−0.64	0.73	***0.93***					
8. SC	3.15	1.05	0.17	0.77	0.71	0.84	0.41	0.80	−0.76	0.46	0.63	***0.88***				
9. PV	2.90	1.07	0.07	0.77	0.73	0.69	0.66	0.59	−0.63	0.64	0.82	0.63	***0.81***			
10. FE	2.64	1.00	0.26	0.74	−0.40	−0.53	−0.23	−0.45	0.50	−0.25	−0.34	−0.48	−0.30	***0.79***		
11. EI	2.90	1.10	0.03	0.86	0.81	0.82	0.56	0.70	−0.79	0.58	0.76	0.76	0.76	−0.47	***0.77***	
12. ST	3.23	1.83	0.51	0.79	−0.46	−0.49	−0.32	−0.40	0.51	−0.35	−0.43	−0.43	−0.41	0.40	−0.46	***0.77***

The bold italic diagonal values represent the square root of AVE.

### 4.3 The relationship between mobile social media functions, exercise identity, and social-media-specific sedentary time

In the AMOS 20.0 software, when the sample size is larger than 200, it usually leads to an excessively large chi-square value, resulting in poor fit. Therefore, the fit value needs to be corrected by Bootstrap (27) The Bollen-Stine Bootstrap correction results for the model fit are as follows: χ2 = 3412.92, χ2/df = 2.55, df = 1,339, GFI = 0.94, AGFI = 0.94, CFI = 0.95, NFI = 0.95, TLI = 0.95, RMSEA = 0.04. All the fit values in this study passed the test, indicating that the results of this study model are acceptable. According to the results in Table 4, the total direct effect γ (non-standardized coefficient) of the mobile social media function in predicting the sports identity is 0.72, *Z* = 2.80 (p-corrected = 0.36 > 0.05), and H1 is not supported, indicating that the mobile social media function has no significant positive predictive effect on the sports identity. However, further analysis reveals that IR, GU, and SC have a significant positive predictive effect on EI; VM has a significant negative predictive effect on EI; and AA, PS, GS, and RE have no significant predictive effect on EI.

**Table 4:** Path analysis table for structural equation.

Hypothesis	Path	Point estimate	Product of coefficients		95% CI		*p*	p correction	Judgment
		γ	σ	Z	Lower Limit	Upper Limit			
H1	Direct Total Effect	0.72	0.26	2.80	−0.20	0.94	0.15	0.36	Not supported
H1a	IR → EI	0.20	0.03	6.06	0.14	0.27	0.001	0.02	supported
H1b	GU → EI	0.38	0.11	3.32	0.08	0.57	0.006	0.03	supported
H1c	AA→EI	0.02	0.03	0.52	−0.05	0.08	0.56	0.75	Not supported
H1d	PS → EI	0.05	0.04	1.21	−0.03	0.12	0.22	0.45	Not supported
H1e	VM → EI	−0.16	0.12	−1.32	−0.57	−0.04	0.01	0.05	supported
H1f	GS → EI	0.03	0.04	0.82	−0.05	0.11	0.37	0.56	Not supported
H1g	RE → EI	0.04	0.04	1.05	−0.04	0.14	0.26	0.49	Not supported
H1h	SC → EI	0.15	0.06	2.44	0.05	0.30	0.006	0.03	supported
H2	Direct Total Effect	0.12	0.43	0.27	−0.55	1.16	0.85	0.87	Not supported
H2a	IR → ST	−0.14	0.11	−1.27	−0.38	0.07	0.19	0.43	Not supported
H2b	GU → ST	−0.15	0.21	−0.72	−0.53	0.30	0.44	0.63	Not supported
H2c	AA→ST	−0.04	0.11	−0.33	−0.26	0.19	0.70	0.83	Not supported
H2d	PS → ST	−0.05	0.14	−0.47	−0.37	0.20	0.57	0.73	Not supported
H2e	VM → ST	0.50	0.21	2.42	0.17	1.01	0.005	0.04	supported
H2f	GS → ST	−0.05	0.14	−0.35	−0.33	0.23	0.70	0.83	Not supported
H2g	RE → ST	−0.12	0.12	−1.00	−0.38	0.13	0.33	0.59	Not supported
H2h	SC → ST	0.19	0.17	1.07	−0.18	0.50	0.35	0.57	Not supported

**p < 0.01, *p < 0.05.

The total direct effect γ (non-standardized coefficient) of the mobile social media function in predicting the specific sedentary time of social media is 0.12, *Z* = 0.27 (p-corrected = 0.87 > 0.05), and H2 is not supported, indicating that the mobile social media function has no significant positive predictive effect on the specific sedentary time of social media. However, further analysis reveals that VM has a significant positive predictive effect on ST; the other functions have no significant predictive effect on sedentary behavior (Figure 2).

**Figure 2:**
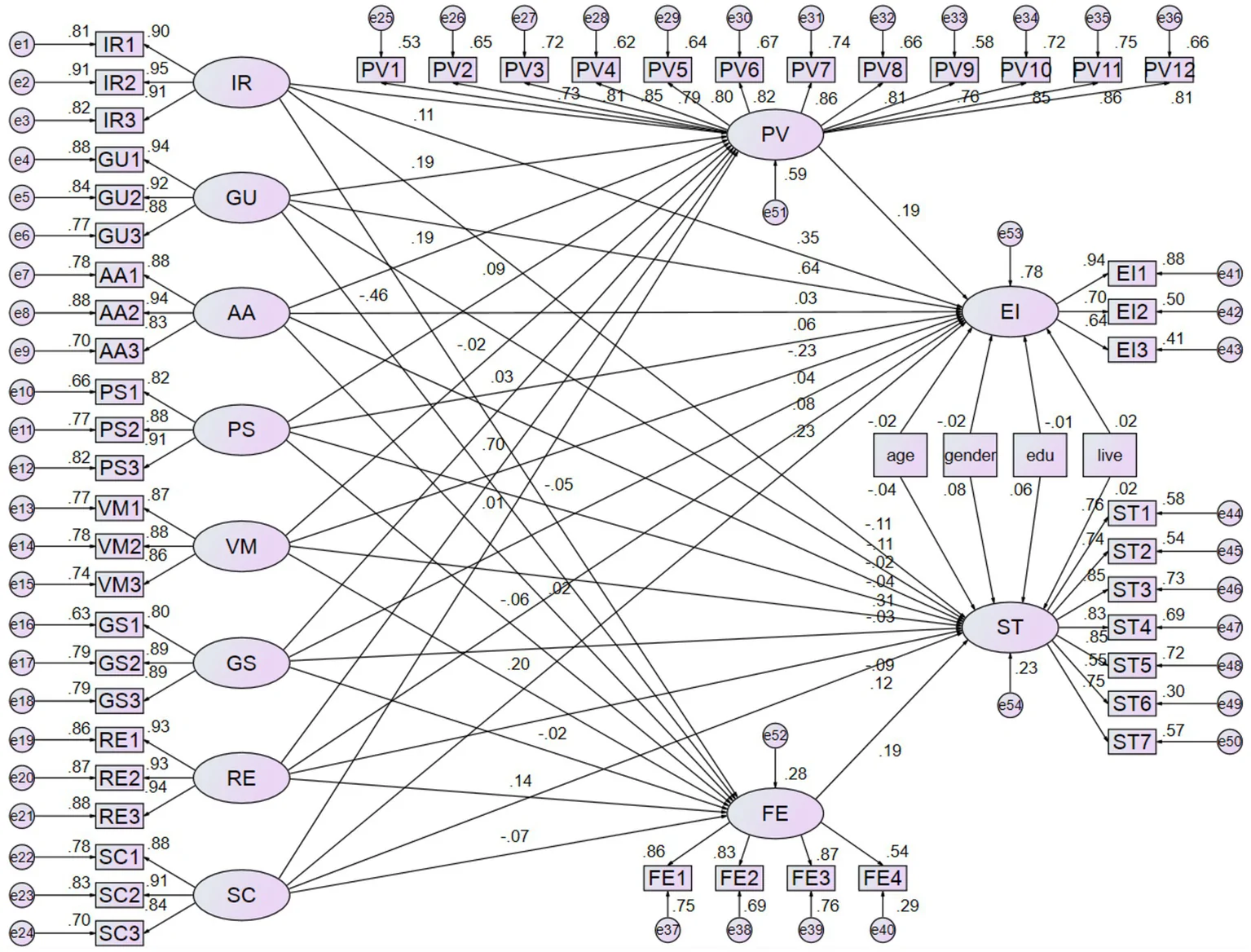
Diagram of structural equation model analysis.

### 4.4 Mediation effect analysis in structural equation model

In the structural equation model, the bias-corrected parameter Monte Carlo bootstrapping method (repeated sampling 5,000 times) is used to test the mediating effect of the model. The results are shown in Table 5. The mobile social media function indirectly predicts EI through PV, with an indirect total effect *Z* = 3.34, 95% CI [0.07, 0.24], p-corrected (padj) = 0.02 < 0.05, f2 = 0.04 (small effect size), and H3 is supported, indicating that the mobile social media function indirectly predicts movement identity through perceived value. Further analysis reveals that AA indirectly predicts EI through PV, with *Z* = 2.56, 95% CI [0.009, 0.05], p-corrected = 0.02 < 0.05; RE indirectly predicts EI through PV, with *Z* = 3.30, 95% CI [0.03, 0.12], p-corrected = 0.02 < 0.05; none of the other functions can indirectly predict EI through PV, indicating that only interaction and recording focus can indirectly predict movement identity through perceived value.

**Table 5:** Analysis of mediating effects of structural equations.

Hypothesis	Path	Point Estimat	Product of Coefficients		95% CI		*p*	p correction	Effect Size	Judgment
		γ	σ	Z	Lower	Upper				
H3		0.15	0.04	3.34	0.07	0.24	0.001	0.02	0.04	supported
	IR-PV-EI	0.01	0.008	1.50	0	0.03	0.05	0.16		Not supported
	GU-PV-EI	0.02	0.01	1.75	0.003	0.05	0.02	0.06		Not supported
	AA-PV-EI	0.02	0.009	2.56	0.009	0.05	0.001	0.02		supported
	PS-PV-EI	0.01	0.009	1.44	−0.002	0.04	0.09	0.24		Not supported
	VM-PV-EI	−0.002	0.01	−0.20	−0.03	0.02	0.78	0.82		Not supported
	GS-PV-EI	0.004	0.009	0.44	−0.01	0.02	0.60	0.74		Not supported
	RE-PV-EI	0.076	0.02	3.30	0.03	0.12	0.002	0.02		supported
	SC-PV-EI	0.001	0.01	0.10	−0.02	0.02	0.92	0.92		Not supported
H4		−0.08	0.09	−0.92	−0.27	0.13	0.22	0.45	0.02	Not supported
	IR-FE-ST	−0.01	0.02	−0.58	−0.08	0.03	0.47	0.65		Not supported
	GU-FE-ST	−0.12	0.06	−2.10	−0.27	−0.02	0.02	0.07		Not supported
	AA-FE-ST	0.007	0.02	0.30	−0.04	0.05	0.74	0.81		Not supported
	PS-FE-ST	−0.02	0.03	−0.71	−0.10	0.03	0.34	0.58		Not supported
	VM-FE-ST	0.06	0.04	1.41	0.006	0.19	0.03	0.05		supported
	GS-FE-ST	−0.006	0.03	−0.21	−0.07	0.05	0.74	0.83		Not supported
	RE-FE-ST	0.04	0.03	1.29	−0.004	0.11	0.10	0.25		Not supported
	SC-FE-ST	−0.02	0.04	−0.62	−0.12	0.03	0.37	0.58		Not supported

* *p* < 0.05.

The mobile social media function indirectly predicts ST through EF, with an indirect total effect *Z* = -0.92, 95% CI [−0.27, 0.13], p-corrected = 0.45 > 0.05, f2 = 0.02 (small effect size), and H4 is not supported, indicating that the mobile social media function cannot indirectly predict specific sedentary time through immersive experience. Further analysis reveals that VM indirectly predicts ST through EF, with *Z* = 1.41, 95% CI [0.006, 0.19], p-corrected = 0.05; the other functions cannot indirectly predict ST through EF, indicating that only video media and personalized algorithms can indirectly predict specific sedentary time through immersive experience.

### 4.5 Sensitivity analysis

To enhance the credibility of the research conclusions, we conducted a model sensitivity test. The sample was randomly divided into two groups, and a multi-group structural equation model was used to test the similarities and differences of the theoretical model between the two randomly selected groups of samples, thereby testing the sensitivity of the model. The measurement weighted model, structural weighted model, structural covariance model, structural residual model, and measurement residual model were set up in an order from loose to strict, and the model fit degrees between each pair were analyzed differently in sequence. As shown in Table 6, the factor loadings of the measurement weighted model were set to be equal for a total of 38 factors, and χ2 increased by 49.55, *p* = 0.10, indicating that the factor loadings of all 38 factors were equal; on the basis of restricting the measurement weighted model, 42 structural model path coefficients were further restricted, χ2 increased by 45.16, *p* = 0.34, indicating that the 42 structural model path coefficients were equal; on the basis of restricting the structural model, 12 structural model covariances were further restricted, χ2 increased by 4.95, *p* = 0.96, indicating that the 12 structural model covariances were equal; on the basis of restricting the structural model covariances, 4 structural model residuals were further restricted, χ2 increased by 8.26, *p* = 0.08, indicating that the 4 structural model residuals were not equal; on the basis of restricting the structural model residuals, 50 measurement model residuals were further restricted, χ2 increased by 157.63, *p* < 0.001, indicating that the 50 measurement model residuals were not equal; however, ΔNFI, ΔIFI, ΔRFI, and ΔTLI were all < 0.05, ΔCFI < 0.01. All results were in line with Kline's standards (28), indicating that the research model did not have significant differences between the two randomly selected groups of samples, the model was stable, and the model passed the sensitivity test.

**Table 6:** Model sensitivity test (n_1_ = 221; n_2_ = 231).

Model	Δ*df*	Δχ^2^	p	ΔNFI	ΔIFI	ΔRFI	ΔTLI	ΔCFI
Measurement weights	38	49.55	0.10	0.002	0.002	−0.003	−0.004	0.00
Structural weights	42	45.16	0.34	0.001	0.002	−0.004	−0.004	0.00
Structural covariances	12	4.95	0.96	0.00	0.00	−0.001	−0.001	0.00
Structural residuals	4	8.26	0.08	0.00	0.00	0.00	0.00	0.00
Measurement residuals	50	157.63	0.00	0.005	0.006	−0.001	−0.001	−0.004

## 5 Discussion

### 5.1 Functional specificity of mobile social media features

This study examined the associations between mobile social media features and two outcomes in older Chinese adults: exercise identity and platform-specific sedentary time. The analysis was guided by the S-O-R framework. Unlike prior work that treats social media use as a single construct (27), we disaggregated specific functions. The results showed that the overall direct effect was not significant. However, the pattern differed across functions. Information retrieval, online guidance, and social comparison were positively associated with exercise identity. Video media and personalized algorithms were negatively associated with exercise identity. They were also positively associated with platform-specific sedentary time. This pattern suggests a “dual-risk” profile.

This differentiation can be understood through functional heterogeneity. Information retrieval, online guidance, and social comparison share instrumental and goal-oriented features. They provide cognitive support and social validation for constructing the “exerciser” self-label through knowledge provision, skill demonstration, and social feedback (4, 5). This is consistent with identity-based behavior theory. When a behavior carries both personal meaning and social recognition, it is most readily integrated into the self-concept (14). For older adults undergoing retirement role transitions, adopting an exercise identity may serve as a compensatory social role. It may help maintain self-worth and social connectedness (29).

In contrast, video media and personalized algorithms are designed around immersive consumption. Their variable reward and auto-play mechanisms tend to induce flow states. This blurs time boundaries and weakens self-regulation (6, 30). As a result, limited time budgets shift from physical exercise to screen-based sitting (31). Functional heterogeneity also explains why the total direct effects for H1 and H2 were not significant. The positive associations of instrumental functions were statistically offset by the negative associations of video algorithms. This null total effect suggests that intervening on social media use as an undifferentiated construct may fail due to the mutual cancelation of heterogeneous sub-effects. Precision efforts should target specific functions rather than overall usage duration (32).

### 5.2 Differentiated mediating mechanisms: perceived value versus immersive experience

The mediation results provide key clues for understanding functional differentiation. Perceived value played an overall mediating role between mobile social media features and exercise identity. This mediating effect was functionally selective. Only two functions-attention and interaction, and recording-showed indirect associations through perceived value. This selectivity is consistent with the findings of mediation model studies based on the S-O-R theory. In mobile short-video apps, perceived value and immersive experience jointly mediated the effect of media interaction on consumption intention. However, the psychological mechanisms underlying these two mediators differ fundamentally (8).

Perceived value centers on users’ overall utility evaluation after weighing gains and losses (4). Its activation depends more on emotional and social feedback than on information acquisition alone. This aligns with Yerrakalva and colleagues’ systematic review of mobile health interventions. The review noted that mHealth app effectiveness depends not only on information provision but also on whether users perceive the platform experience as a “valuable” health management tool (14). Similarly, Yerrakalva and colleagues’ meta-analysis of smartphone apps for exercise identity promotion found that apps integrating goal-setting, self-monitoring, and social sharing features were more likely to drive behavior change by enhancing users’ intrinsic value perception (33).

In contrast to the overall mediating effect of perceived value, the overall indirect effect of immersive experience between mobile social media features and social-media-specific sedentary time was not statistically significant. However, for video media and personalized algorithms specifically, immersive experience did mediate the association with sedentary time. This finding can be explained by flow theory. Csikszentmihalyi and LeFevre’s classic work characterized flow as an optimal immersive experience. Its features include intense concentration, distorted time perception, and temporary loss of self-awareness (34). Algorithmic recommendation systems on short-video platforms use auto-play, infinite scrolling, and variable rewards. These design elements exploit this psychological mechanism to maximize user engagement (35).

Hall and Liu’s research on social media use and social displacement also suggests that immersive platform designs may reallocate time resources from exercise identity to screen consumption through a “time displacement” mechanism (36). However, the overall mediation effect of immersive experience was not significant in this study. The key reason is that not all mobile social media functions induce flow states of equal intensity. Functions such as information retrieval and online guidance have clear goal boundaries and task endpoints. The immersion they trigger ends naturally when the goal is achieved. Only the flow driven by video media and personalized algorithms lacks natural stopping cues. It is therefore more likely to translate into prolonged sedentary time (6).

This finding has two implications for digital health promotion in older adults. On one hand, platform design should strengthen functional modules that reinforce exercise identity through perceived value. For example, linking exercise records with family social networks may enhance emotional and social value through social feedback (5). On the other hand, age-friendly design for video media should introduce “anti-flow” mechanisms. These include periodic standing reminders and session time interruptions. Such features can disrupt algorithm-driven immersive consumption loops (6, 37).

### 5.3 Practical implications

This study offers several implications for age-friendly digital health interventions. First, given the positive associations of information retrieval, online guidance, and social comparison with exercise identity, and the unique risk of video media and personalized algorithms for sedentary time, treating social media use as a homogeneous construct and imposing uniform screen time limits may reduce intervention effectiveness by suppressing beneficial functions. Differentiated design strategies are therefore more appropriate.

Specifically, three approaches are worth considering. For algorithm-driven video feeds, customizable standing reminders and session interruption mechanisms could be embedded. These would interrupt the temporal perception of flow states and reduce sedentary risk without forcing users to leave the platform. Goal-setting and activity recording functions could be integrated into family social network interfaces. This would leverage kinship structures in the Chinese context to enhance social commitment and perceived value, reinforcing exercise identity internalization through existing relationship channels. Algorithm diversification strategies could also be implemented. Health-promoting content could be periodically interweaving into entertainment-oriented feeds. This would mitigate filter bubble effects and increase older adults’ exposure to exercise-related stimuli.

Second, traditional digital literacy programs for older adults have largely focused on reducing overall usage time. However, this study suggests that the root issue is not use per se, but functional choice. Digital literacy education should therefore be upgraded to functional literacy training. Older users should be guided to identify and prioritize functions that support health identity construction, such as information retrieval and online guidance. They should also be made aware of immersive functions that may inadvertently extend sedentary time. Community-level interventions could benefit from recruiting older digital opinion leaders within social networks to model and normalize the use of health-promoting platform features.

Third, platform intervention design must be understood within the Chinese cultural context. In China, older adults’ mobile social media use is deeply embedded in family-oriented social networks. The dominant platform WeChat, for example, serves primarily to maintain intergenerational kinship ties. Health-related self-presentation in this context carries not only personal meaning but also a signaling function—conveying positive coping with aging to adult children.

### 5.4 Limitations and future directions

This study has several methodological limitations. First, the cross-sectional design precludes causal or directional inference. The observed associations between mobile social media features, exercise identity, and social-media-specific sedentary time cannot establish temporal order. Reverse causality is highly plausible. Older adults with stronger exercise identity may actively self-select fitness-related platform functions such as goal-setting and online guidance. This could inflate the observed positive associations. Conversely, older adults with longer social-media-specific sedentary time may be algorithmically directed toward video content pools. This could create a self-reinforcing cycle. Such reverse effects may simply reflect pre-existing behavioral patterns rather than being caused by platform functions.

In addition, although we controlled for demographic covariates including age, gender, education, and living arrangements in the structural model, unmeasured variables may still confound the results. These include pre-existing chronic health conditions, physical functional limitations, and personality traits. Such factors could simultaneously influence both the tendency to use specific platform functions and the outcomes of exercise identity and sedentary time, thereby affecting internal validity.

Second, measurement limitations should be acknowledged. All constructs were measured via self-report. This may introduce social desirability bias and recall bias. The exercise identity scale measures self-perception and social recognition rather than objective exercise identity behavior. Although exercise identity is a confirmed predictor of long-term behavioral maintenance, the lack of behavioral measures such as accelerometry limits our ability to draw inferences about actual exercise identity behavior.

Third, sample representativeness is limited. This study used a non-probability sampling strategy combining purposive geographic stratification and snowball recruitment. This restricts the generalizability of the findings to the broader Chinese older adult population. Although we sought to cover regional diversity within Shandong Province, the findings cannot be extended to the overall Chinese older adult population or other national contexts without further validation.

Future research could address these limitations in several ways. Longitudinal tracking designs or natural experiments could clarify temporal relationships between social media functions and health outcomes while controlling for time-invariant confounders. Ecological momentary assessment (EMA) could be introduced to capture real-time functional activation and immediate psychological state changes in naturalistic usage contexts, allowing more precise mapping of dynamic transmission chains. Identity measures could be combined with objective exercise identity assessments to bridge the intention-behavior gap. Multi-stage cluster sampling could be adopted to improve sample representativeness across larger geographic areas. Cross-cultural comparative perspectives could also be incorporated. Testing the same functional structure model in different cultural contexts would help determine whether the observed functional heterogeneity is a universal feature of digital platforms or a phenomenon specific to the Chinese context, providing more culturally sensitive evidence for global age-friendly digital health platform design. Additionally, evidence from clinical neuroscience suggests that the impact of different social media functions on cognition and behavior in older people may involve neuroplasticity processes. The synergistic effect of repetitive transcranial magnetic stimulation in alleviating cognitive impairment and negative symptoms associated with schizophrenia is one example of this. Future research needs to incorporate multimodal intervention studies into its exploration of digital health behaviors (38, 39).

## 6 Conclusion

This study is based on the S-O-R theoretical framework. By deconstructing the functional heterogeneity of mobile social media, it reveals the differentiated impacts of these platforms on the movement identities of Chinese aged population and their specific sedentary behaviors. The study found that information retrieval, online guidance, social comparison, and a stronger movement identity are concurrently associated, while the personalized algorithm-driven video function is concurrently associated with a weaker movement identity and prolonged specific platform sedentary time. Perceived value plays a mediating role in the path of social expressive function, and immersive experience plays a mediating role in the algorithm function path of the personalized algorithm. These findings challenge the conceptualization of mobile social media usage as an undifferentiated construct in health research, and provide evidence-based evidence for the design of empowering rather than deprivative aged-friendly digital environments. It offers a solid scientific basis for formulating effective health intervention strategies for the older adults and guiding healthy lifestyles.

## Data Availability

The raw data supporting the conclusions of this article will be made available by the authors, without undue reservation.
